# Basic Psychological Needs in the Face of Achievement Vulnerability: A Study in Young Team Athletes

**DOI:** 10.3390/bs14080697

**Published:** 2024-08-11

**Authors:** Mirella Triguero Martín, David Manzano-Sánchez, Manuel Gómez-López, Juan González-Hernández

**Affiliations:** 1 Department of Personality, Evaluation and Psychological Treatment, Faculty of Psychology, University of Granada, C/ Beiro, s/n, 18011 Granada, Spain; mirella15@correo.ugr.es (M.T.M.); jgonzalez@ugr.es (J.G.-H.); 2 ACAFYDE Research Group, Faculty of Psychology and Education, University of Extremadura, Av. De Elvas, s/n, 06006 Badajoz, Spain; davidms@unex.es; 3 Department of Physical Activity and Sport, Faculty of Sport Sciences, University of Murcia, C/ Argentina, s/n, 30720 Murcia, Spain

**Keywords:** perfectionism, fear of failure, basic psychological needs, young athletes, achievement vulnerability

## Abstract

The susceptibility of athletes to experience of emotional and psychological difficulties arising from the pressure and expectations associated with achieving and maintaining high performance can become a vulnerability in the desire to achieve success in sport. This study aims to investigate the protective value in the perception of satisfaction in basic psychological needs against the vulnerability that perfectionism generates in the appearance of reactivity linked to fear of failure. A cross-sectional, relational, and semi-randomized research design was used, applying perfectionism, fear of failure, and basic psychological needs measures adapted to both the competitive sports context and the Spanish language in a sample of 372 young Spanish athletes, under descriptive analyses and predictive models. The results showed that as the age of the participants increased, the indicators of perfectionism and fear of failure decreased, with no gender differences. The results offer and confirm the positive relationships between the dimensions of perfectionism and fear of making mistakes (where processes such as self-devaluation and fear of failing the people that participants deemed as important to them are intertwined). The perception of satisfaction of the basic psychological needs of autonomy, social relationships, and competence emerges as protective factors that mediate the perfectionism–fear of failure relationship. On the other hand, discrepancies are shown between the perfectionist dimensions concerning the relationships with the BPNs, describing certain sources of vulnerability, although there are adjustments of mental effort and discomfort in the young athletes. The conclusions offer the opportunity to investigate the aspects that facilitate the emergence of fear of failure in young athletes, mainly the performance of coaches connected to the emergence of patterns in pursuit of perfection.

## 1. Introduction

Adolescence is a decisive stage in which young people acquire and consolidate their lifestyles [1], encouraging the practice of beneficial activities (sports practice), seeking to improve internal psychological processes (e.g., enhancing self-confidence and self-esteem) [2,3] or external ones (e.g., social relationship skills) [4,5]. A large body of research shows not only that sporting success depends on physical skills but that psychological factors are essential for the balance between effort, demand, and perceptions linked to well-being and adaptation in grassroots sports [6,7].

Performed on a regular and competitive basis, sport and the relentless pursuit of achievement goals expose the young athlete to demand and coexistence with situations of error typical of any adaptation and learning process, although often misinterpreted as failures [8]. Such experiences impact athletes’ internal processes, (e.g., guilt, shame, distress) that become a specific vulnerability in achievement [9] for those with perfectionist patterns. On the other hand, we found that interpersonal relationships in a sports team involve interdependence among team members, conditioning the collective performance that can be altered by any vulnerability process of any of its members [10,11]. In different systematic reviews, it is evidenced that practicing collective sports at early ages enhances the construction of social identity and connection as a potentiating source of perceived social support, the construction of social identity, and the protection of social isolation that precedes depressive or anxious episodes [12,13].

### 1.1. Fear of Failure at the Top of the Perfectionist Iceberg

Perfectionism is a multidimensional construct that maintains maturational development during the adolescent stage [14]. It is described as a transdiagnostic trait of different psychological disorders based on the presence of desires to reach high standards in performance and achievement, coupled with negative cognitive tendencies (e.g., uncertainty, doubts in executions and decisions) towards that which implies not reaching such ideals (e.g., negative self-judgments or judgments of other recognition figures, shame) [15,16]. In classical models [17,18,19], perfectionism has been understood as a trigger and maintainer of the fear of making mistakes, identifying it as the tendency to perceive as threats or cognitive–emotional instability the mismatch between what is expected (significant personal goals) and what is achieved (considered as failure) [19,20]. The most maladaptive thoughts are framed in the pattern of what is understood as socially prescribed (e.g., “it is what everyone would do in my situation”, “it is the only way to feel respected and accepted”, “it is what everyone expects of me”) [21].

Flett and Hewitt [17] describe three dimensions of perfectionism: self-oriented, socially prescribed perfectionism, and other-oriented. The most maladaptive thoughts fall into the pattern of what is understood as socially prescribed (e.g., “it is what everyone would do in my situation”, “it is the only way for me to feel respected and accepted”, “I am not able to live up to what is expected of me”) [21,22], which, coupled with self-oriented (e.g., “I must always be the best at everything” or “I want to achieve the most”), cause intense sources of discomfort, mainly on the way to achievement or where adequate performance has to be shown (e.g., school, sport).

Considered then with negative connotations from clinical perspectives [18,19], studies in sport contexts have shown that perfectionism could be “positive or adaptive” in terms of the utility it offers (mainly in the short term) for achievement and the aspects linked to successful achievement (e.g., self-efficacy) [15,16,23,24]. From this point of view, perfectionism awakens in the athlete a positive attentional focus (perfectionist effort) as they feel close to the goal for which they are striving. At the same time, personal and interpersonal concerns (perfectionist worries) also emerge, through which the athlete experiences cognitive (e.g., ruminations, feelings of loss of identity) and behavioral (e.g., eating problems, insomnia, or conflicts with others) maladjustments for not reaching the perfection they expect or losing the perfection they have already achieved [24,25].

However, longitudinal studies have shown that athletes with more perfectionist tendencies and those who maintain such efforts and concerns over a longer period develop maladaptive processes that make them vulnerable and wear them out psychologically, physically, and socially [9,14]. Prolonged and high-intensity imposition of high levels of excellence [26] involves great pressure and the appearance of concerns about not being perfect or not failing themselves or others, directly or indirectly affecting performance and mental health (e.g., depression, anxiety, burnout) [25,27].

Stoeber [16] linked extrinsic motivation with vulnerability and intrinsic motivation with motivational strengths, well-being, and performance enhancement. Other research focused on athletes has considered a functional part of perfectionism when it is positively associated with aspects such as self-confidence, self-efficacy, planning, and persistence [27], while usually the phenomena around perfectionism are considered dysfunctional and linked to uncertainty regarding fear after performance, anxiety about not being able to perform effectively, avoidance of public exposure when perfection is not achieved, and ultimately fear of failure [28].

Stoeber et al. [29,30,31] (original version) conducted versions of the MIPS (Multidimensional Inventory of Perfectionism in Sport) to measure the dimensions of perfectionism adapted to the sport context from a two-dimensional perspective. They considered Perfectionist Efforts (efforts made by athletes in generating expectations, designing and planning how to achieve them, dedicating high levels of time and energy) and Perfectionist Concerns (negative cognitive–emotional reactivity to doubts about the achievement of the pursued goals, magnification of external pressures, fears about the consequences of failure) and measured from different perspectives (of the athlete, parents, coaches). Subsequently, Pineda-Espejel et al. [32] adapted to Spanish the scale focused on athletes about themselves (Short Scale of Multidimensional Perfectionism in Sport).

Given that sport is an environment in which athletes compare themselves and experience perceptions of incompetence when they make mistakes in front of others (e.g., coaches, rivals, teammates) [22,26,30,33,34,35], fear of failure may then appear when they compare themselves or others to themselves in the past or in their internalized projection (even if others or themselves have made other, more serious mistakes) [11,19,34]. In more competitive sporting environments, the personal desire to achieve goals and the cognitive uncertainty of being able to achieve them trigger the fear of making mistakes, and this is altered in its subjective interpretation by aspects such as whether that mistake is public (in front of others or in front of significant others) or does not conform to their expectations of achievement (e.g., “trying hard should cause me to make fewer mistakes”) [36,37]. For the athlete, fear of making mistakes is then described as a subjective emotion, as a state of mind or feeling that has environmental antecedents (e.g., external pressure) and behavioral consequences (e.g., fear of failing others, being alienated, experiencing embarrassment or devaluing yourself). Despite being a crucial aspect in the psychological functioning of athletes, and linked to achievement orientation, it is a concept not greatly explored in sport psychology [37,38].

In team sports, the effects of perfectionism on the fear of failure are very present, as mistakes and deficiencies in sports actions can harm someone beyond oneself [39], increasing the processes of undervaluation for failing other people, full of negative connotations and affecting self-esteem. However, in team sports, there are more immediate socio-cognitive repercussions (e.g., “perceiving the harm to teammates of my failure”). The phenomenon of fear of making mistakes is individual, a process centered on the person, regardless of whether individual or team sports are practiced. The strength of the perceived social valuation (e.g., perceived technical feedback, social approval from peers), or rather the fear of losing it, is one of the sources of concern in athletes [40,41,42,43] that reinforces the destructive paths that begin with the tendencies to achieve perfection in sports actions.

### 1.2. NPB Satisfaction as a Positive Resource in Young Athlete Performance

Basic psychological needs (BPNs) [44] are described as psychological resources centered on the satisfaction and development of competence (perceived ability and sense of mastery to perform tasks with different levels of difficulty), autonomy (ability to feel responsible for one’s behavior or to perform actions of one’s own free will), and relatedness to others (feeling connected, supported or loved by other people). Integrated into the Self-Determination Theory (SDT) [45,46,47,48,49], the perception of satisfaction with BPNs facilitates optimal psychological development and state of personal well-being [50], as well as an improvement of those aspects referred to performance and efforts to achieve internalized goals [51,52].

Adapting such description to sports contexts and living with the satisfaction of BPNs connects with intrinsically motivated sports behaviors (e.g., effort, participation, pride, and identification) [53,54]. Conversely, the feeling of frustration elicits distress and adaptive maladjustment processes (e.g., fear, anxiety, or exhaustion) [55]. Therefore, when the sports experience provides an environment that facilitates relationships with others, uplifting values friendship and bonding (relationship with others), allows the possibility of choice (autonomy), and the feeling of adequate practice (competence) [26,56], athletes show greater intrinsic motivation, feel greater pride linked to the effort to improve (and less shame to make mistakes in that same process), and show a more solvent management of situations involving external pressure [57,58].

In this sense, the perception of satisfaction with the NPBs has been related as a protective factor against maladaptive responses [45] in athletes such as anguish [59], anxiety [60], depressive episodes [58], or related responses such as fear of failure (for example, devaluation for not reaching self-imposed performance standards) [60,61,62,63].

### 1.3. Objectives and Hypotheses

**Figure 1:**
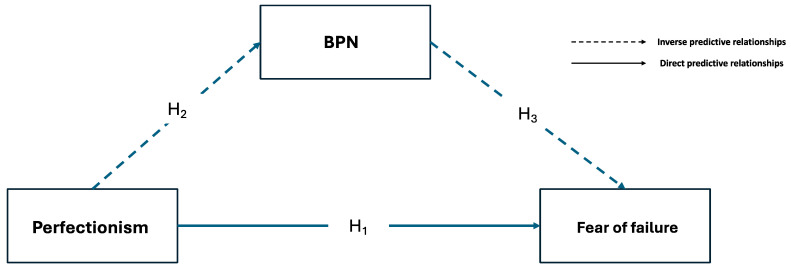
Hypothetical model.

Following the reviewed literature, the present paper aims to explain how perceived satisfaction with BPNs mediates the relationship between perfectionism and fear of failure in a sample of young athletes playing team sports. Based on this, the hypothesized model (see Figure 1) expects to find that perfectionism will show a direct and positive relationship with fear of making mistakes (H_1_), while the mediation of satisfaction with BPNs will exert an indirect and protective effect on the negative effects of perfectionism (H_2_) and reduce the fear of making mistakes (H_3_).

## 2. Materials and Methods

### 2.1. Design, Participants, and Procedure

The methodological procedure is described under the STROBE Statement—checklist of items that should be included in reports of observational studies (https://www.strobe-statement.org/checklists/), accessed on 5 June 2024. A cross-sectional, quantitative design with semi-random sampling (participants were not directly selected at random and belonged to the clubs contacted) and mediated prediction analysis was used. Considering, through a power analysis [64], an expected sample of 341 athletes, the following results were obtained using a sample of 372 young people practicing different team sports, with M_age_ = 16.72 years (SD = 3.59; range 14–19 years old) of which 52.15% (*n* = 194) were boys and 47% (*n* = 178) were girls. Table 1 shows data collected from different handball, soccer, volleyball, and basketball sports clubs in different parts of the country.

The procedure involved the initial preparation of a list of collective sports clubs (soccer, volleyball, basketball, and handball) that had teams aged between 12 and 19 years (infantile to juvenile stages). Next, a general letter was drafted, briefly explaining the objective of the study and the collaboration of players and coaches, which was sent to clubs via email or Instagram. Through video calls, the individuals who agreed to participate were contacted and asked to pass an informed consent document to legal guardians (parents/parents/coaches) according to the protocol approved by the Ethics Committee of the University of Granada (ID:1494/2017), following the guidelines of the Declaration of Helsinki [65]. After that, those who accepted (mainly coaches or club managers) were presented with a link with the battery of items to be distributed among their athletes, with an explanatory text on the expected objectives, how to complete it, and the time required. In addition, the document explained that the information provided is anonymous, also clarifying to them that the use of it is for scientific purposes only.

### 2.2. Variables and Instruments

Perfectionism: The Spanish adaptation [32] of the Multidimensional Perfectionism in Sport Scale-2 (Sport-MPS-2) [31] composed of 10 items was used, specifically describing two factors: Striving for Perfection (e.g., “I strive to be as perfect as possible”) and Negative Reaction to Imperfection (e.g., “I get furious if I make mistakes”). Responses were provided on a Likert-type scale from 1 (never) to 6 (always). Alpha Cronbach obtained was 0.87 and CFA description [χ^2^ = 482.85; *p* = 0.00; χ^2^/gl = 3.73; CFI = 0.92; IFI = 0.90; TLI = 0.92; RMSEA = 0.06, SRMR = 0.04] showed robust.

Fear of failure: The Spanish sample adaptation [66] of the Fear of Failure Inventory (PFAI-S) [36] was used. Composed of 25 items, it considers the global factor “Fear of failure” (e.g., “when I make a mistake, it upsets the people I care about”, “when I am not successful, some people are not interested in me”). Responses were provided on a Likert-type scale from 1 “I don’t believe it at all” to 5 “I believe it 100%”. The overall internal consistency (alpha) was 0.94, and the CFA showed and fit the internal structure of [χ^2^ = 615.03; *p* = 0.00; χ^2^/gl = 3.02; CFI = 0.91; IFI = 0.92; TLI = 0.93; RMSEA = 0.07, SRMR = 0.03].

Satisfaction of Basic Psychological Needs: The Basic Psychological Needs Scale (BPNES) [67], in the version adapted to Spanish by Pineda-Espejel et al. [68], was administered. It includes 12 items divided into three dimensions to assess Autonomy (α = 0.83; e.g., “The training program I follow fits my interests”), Competence (α = 0.79; e.g., “I have had a great progression concerning the pursued result”), and Relation with others (α = 0.92; e.g., “I feel very comfortable when I exercise with other athletes”). Responses were provided on a 5-point Likert scale ranging from 1 (strongly disagree) to 5 (strongly agree). The general internal consistency was 0.94 and the CFA described an internal structure to be adequate [χ^2^ = 248.36; *p* = 0.00; χ^2^/gl = 2.38; CFI = 0.93; IFI = 0.92; TLI = 0.90; RMSEA = 0.07, SRMR = 0.02].

### 2.3. Data Analysis

Before testing the hypotheses, descriptive, normality, reliability, and central tendency measures (K-S, means, standard deviations, and reliabilities of the measures) were performed. Next, differential tests (Student’s *t*-test and ANOVA) were performed to consider the influence of categorical variables, and Pearson’s correlation analysis was performed to show linear relationships. Subsequently, the thematic measurement model and the hypothesized structural model were tested using Mplus 7.4 (Los Angeles, CA: Muthén & Muthén) [69]. The fit of the measurement model was checked before testing the structural model. Correlation coefficients between latent variables and factor loadings between each latent variable and its indicators were also examined. The fit of the hypothesized structural model (partially mediated model) was then tested and compared with alternative models (fully mediated models) using chi-squared tests. Using dummy variables, introducing bootstrap confidence intervals (sample > 5000) corrected for bias for specific indirect effects, a significant effect was considered to exist if the CIs did not include zero [70].

Several fit indices were used to assess model fit, including the chi-square (χ^2^) statistic, the Tucker–Lewis index (TLI), the comparative fit index (CFI), and the root mean square error of approximation (RMSEA). Although the χ^2^ statistic tests whether the actual data fit the model, it is sensitive to sample size, with the cutoff value for RMSEA (<0.08) [71], TLI (>0.95), and CFI (>0.90–0.95) [72]. The initial model included all hypothesized paths between the latent variables. In the second model, we restricted a direct path between the dimensions of perfectionism and fear of failure, and in the final model, we restricted the dimensions of perfectionism to BPNs. In testing the hypothesized model, we aimed to test whether the direct effects of the dependent (perfectionistic strivings and negative reactions to imperfection), independent (fear of failure), and mediating (BPN) variables showed significant relationships and whether these relationships were fully or partially mediated [73].

## 3. Results

### 3.1. Descriptive Analysis

The descriptive values, normality fit correlations, and internal consistency of the variables used are shown in Table 2. After assessing the multivariate normality of the variables, compliance with the multivariate normality criteria (skewness < 2.0, kurtosis < 7.0) was observed [74]. Differential tests showed no significant differences in gender (Student’s *t*-test), while significant differences were observed in perfectionism (Soccer; F = 6.24; *p* < 0.01; d = 0.18), NPBs (Handball; F = 9.19; *p* > 0.001; d = 0.06), and fear of failure (Handball; F = 3.48; *p* > 0.001; d = 0.31). Regarding years of sport experience, significantly more major differences were found in both perfectionism subdimensions with 2 to 5 years of experience (F = 11.03; *p* > 0.001; d = 0.41) and fear of failure in those with more than 5 years of experience (F = 14.03; *p* > 0.001; d = 0.36).

Striving for Perfection showed strong relationships with Negative Reactions to Imperfection (r = 0.46) and Fear of Failure (r = 0.51), and weak relationships with Relatedness (r = 0.13). Striving for Perfection showed positive and moderate relationships with Autonomy (r = 0.36) and Competence (r = 0.28), and inverse (although weak) with Relation (r = −13). Negative Reactions to Imperfection showed inverse and strong lineal relationships—Autonomy (r = -0.56), Competence (r = −0.64), and Relation (r = −0.48). Fear of Failure showed strong inverse relationships with the BPNs [Autonomy (r = −0.41), Relatedness (r = −0.38), and Competence (r = −0.45)].

### 3.2. Confirmatory Analysis

Robust maximum likelihood estimation [72] was performed and model fit was checked using the scaled χ^2^ (S-B χ^2^) [75] corrected for non-normality, together with the TLI, CFI, and RMSEA fit indices. Subsequently, confirmatory factor analysis was performed for a full measurement model, including all latent variables showing an acceptable fit to the data: S-B χ^2^ (93) = 372 (>5), TLI = 0.96, CFI = 0.97, and RMSEA = 0.03. Correlations between latent variables were shown to be high evidencing the inexistence of multicollinearity (variance inflation factor = 2.00).

### 3.3. Correlation Analysis (Controlling for Gender, Type of Sport, and Experience) and Mediation Effects

The analysis of partial correlations (see Table 3) reveals positive and significant linear relationships between perfectionism and fear of failure. In both controlling types of sport and sporting experience, the variable behavior was similar and significant. While the relationships between Striving for Perfection were positive with Autonomy, Competence, and Fear of Failure, the relationship with Social Relation was negative. On the other hand, Negative Reactions to Imperfection showed negative and strong relationships with Autonomy, Competence, and Social Relation, while the relationships with Fear of Failure were positive.

**Figure 2:**
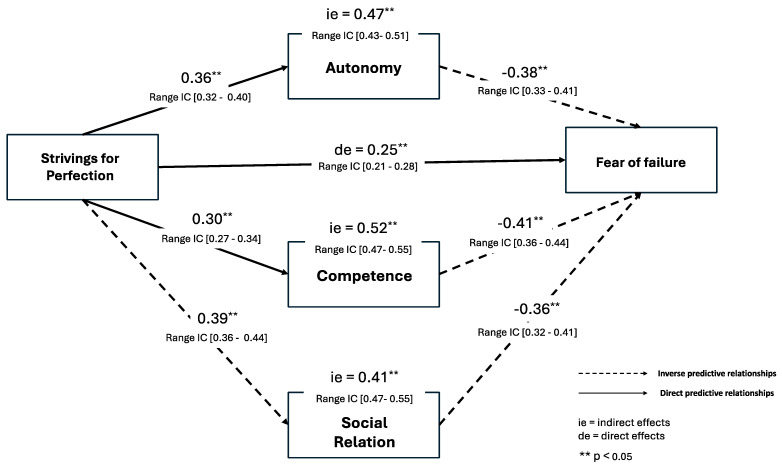
Model Standardized by Striving for Perfection.

**Figure 3:**
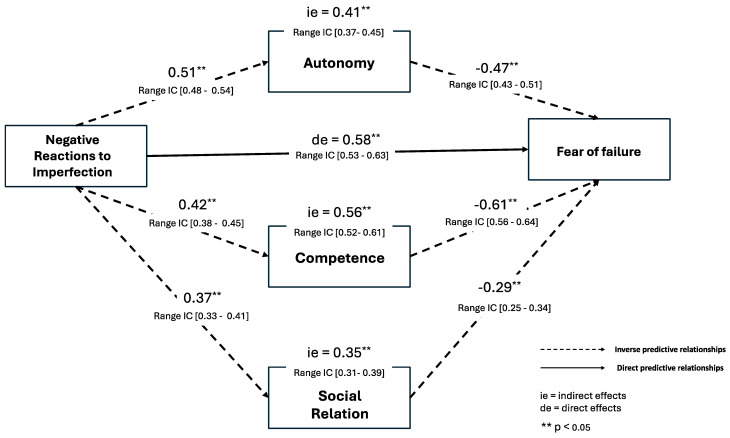
Model Standardized by Negative Reactions to Imperfection.

The results confirmed that BPNs mediate the relationship between perfectionism and fear of failure (β = 32.4; *p* < 0.01), with a significant indirect effect (β = 0.18; *p* < 0.01). The mediation model test also worked for BPNs separately on BPNs (see Figure 2 and Figure 3), with the significant indirect effects to Striving to Perfection [Autonomy (β = 0.47; *p* < 0.01), Competence (β = 0.52; *p* < 0.01), and Social Relation (β = 0.41; *p* < 0.01)] and to Negative Reactions to Imperfection [Autonomy (β = 0.41; *p* < 0.01), Social Relation (β = 0.56; *p* < 0.01), and Competence (β = 0.35; *p* < 0.01)].

## 4. Discussion

The present study aimed to describe the relationships between internalized perfectionism, the perception of satisfaction of basic psychological needs, and the fear of failure response in a sample of young athletes in team sports. The proposed hypotheses aimed to confirm the direct and positive relationships between perfectionism and fear of failure (H_1_), as well as the indirect and protective value of the mediation of each of the basic psychological needs when they are perceived as satisfied (H_2_ and H_3_).

As shown in the results, the H_1_ is confirmed both in the total correlations and partial correlations (controlling for possible differences in gender, sport modality, and years of experience) and in the mediation analysis. The scientific literature has shown similar results. Studies such as Conroy et al. [38] or Hill et al. [76] highlighted the fact that the specific beliefs about the consequences of failure underlying different forms of perfectionism impact fear of failure when acting in combination [23,35,36]. Other studies [15,26,32,58] have shown that perfectionistic preoccupation with mistakes, the perception of external pressures (mainly from parents), and the excess of wanting to achieve sporting accomplishments to please others showed a positive relationship with the fear of experiencing shame and embarrassment in achievement actions and putting thoughts of failure (“if I fail I will no longer be important to the team”, “I don’t know if it is worth my effort”) before positive appraisals of the action.

Furthermore, it appears that when controlling for variables that offer some significant difference (type of sport and years of experience), direct relationships between fear of failure contrast with Stoeber and Stoeber [77] who showed that being a perfectionist (overall or on an individual dimension) is not related to experience. However, they also contrast with other studies that highlight links with fear of failure. The results agree with studies that noted higher levels of fear of failure in less experienced athletes [36,78] or that younger athletes showed greater negative reactions to imperfection, poor rapport with peers, and did not feel accepted, worsening their performance [41,79,80].

Concerning the fulfillment of H_2_ and H_3_, referred to the mediating value of the BPNs, the perfectionist profile is protected when these needs are perceived as satisfied. These results reinforce those obtained in studies such as that of Chen et al. [46], which showed the value these needs offered to well-being in different modalities of sports practice, or that of Haraldsen et al. [81], which linked perfectionist tendencies to the frustration with PNBs (antagonistic process to satisfaction according to TAD) both in the short and long term. Moreover, the absence of significant differences with increasing years of experience reinforces the consistency of effects at all defined stages.

While the connections between Striving for Perfection and the perceived satisfaction of the needs for Autonomy and Competence are positive, the links with the perception of the need for Social Relationships are negative in each of the analyses performed. Although we expected that belonging to a team context would mean psychological functionality being directed towards connection with others, this only partially fulfills H2, which shows that an excess of expectations and a magnification of the value of the goal to be achieved is not in connivance with the search for social relations but rather with distancing from others. Studies carried out in other samples of young athletes (although none in the Spanish context) point out the possible social disconnection offered by perfectionist patterns, where importance is given to the personal and social wear and tear involved in the demands made on oneself and others in the pursuit of a sporting goal [22,82,83,84].

In addition, the prolific scientific literature justifying the positive value of satisfaction of BPNs [50] thus fosters other adaptive psychological resources in team sports (e.g., team cohesion [85]; understanding or improvement of coach–athlete relationships, [86]; individual and group self-regulation [87]) that have an impact on reducing the occurrence of fear of failure despite the existence of a strong influence of perfectionist tendencies [23,52], ego orientations [58], or sport pressure [51].

As for limitations, this study has a small sample size, especially for the age range of 18–23 years. In future research, it would be convenient to increase the number of participants in each of the categories. In addition, the number of young athletes depending on the type of sport is not the same, which is also a limitation when comparing results, so it is necessary for future research to have the number of players per team be similar or equal. An obvious limitation of cross-sectional approaches is that it is difficult to determine the temporal (and causal) sequence of events or to determine whether one variable directly influences another. In addition, it should be noted that different effects may be present in cross-sectional approaches that alter the interpretation not only of the study participants, but also of the interpreters of the results (e.g., recall, measurement, or selection biases). The profile of the coaches can also be a limitation (gender, age, perfectionism trends, etc.). It was not considered, making it difficult to analyze exactly which trait of the coach affects the perception of the coach’s pressure on their athletes.

The work described here inspires the possibility of promoting a line of studies on the aspects that protect from vulnerability the achievements with which the athlete feels they build their sports identity. Furthermore, extending this set of variables with others (even in new cross-sectional studies) will allow us to know early on which essential aspects of psychological functioning are vulnerable (and thus be able to begin to identify and train processes that may be aggravated with high levels of sporting demand and perfectionist patterns (e.g., attachment, narcissism, social isolation)). The current proposal is also inspiring for future longitudinal studies based on the evolution of the relationships between variables at different competitive moments.

## 5. Conclusions

Understanding the latent forces involved in the transdiagnostic essence of perfectionistic patterns combines different theoretical and methodological models to explain athletes’ responses to vulnerability [9]. Studying the constructs that might be associated with perfectionism (in precipitating and maintaining it, as well as in understanding and strengthening it) involves useful strategies to understand the whole picture and obtain better scientific evidence [8]. Even so, pathological or disordered behaviors evident of impairment (e.g., eating disorders, depression, addiction), especially in the short term, do not always emerge, hence scientific approaches and formulations must deepen their detection and contemplate the continuity of studies and experimental methodologies to enhance the effectiveness of the explanations.

The present work aimed to explain how perceived satisfaction with BPNs mediates the relationship between perfectionism and fear of failure in a sample of young athletes playing team sports. Vulnerability to achievement implies the susceptibility of people to interpreting results as being negative. Not admitting errors as being part of a process, as a way of learning, or of identifying what has been achieved means living with the agony and the devaluation of oneself and increasing the symptomatology of a perfectionist self-representation that acts comorbidly towards the appearance of any process of psychological vulnerability in athletes. The strength with which the athlete experiences the impacts of the distancing or slowing down of the achievements they pursue and how the context of competitive sport demands their fulfillment are two simultaneous sides of the same coin which require both dispositional psychological strengths (e.g., perseverance, emotional stability, prosocial attitudes) and learned ones (e.g., motivational strategies, self-efficacy, socioemotional skills).

Even when not exhibiting disordered levels of perfectionism as Hewitt and Flett [17] pointed out almost 35 years ago, living with such signals in the day-to-day competitive environment of a team sport is a problem for adaptation.

Team sports exalt an integrative culture of “we all add up” or “we are a family”, albeit with high levels of competitive struggle among its members to occupy positions of prominence (e.g., everyone wants to be a starter and participate for as long as possible).

Team sports locker rooms, dominated by the power given to coaches for decisions, are ambiguously a risk factor for individual patterns of vulnerability (e.g., comparison, rivalry, and distrust) among team members, immediate performance opportunities and public exposure, need for rapid cognitive–emotional recovery processes in the face of mistakes and protective factors in that they provide social support resources (e.g., cohesion, prosocial and social connection behaviors, performance references, and competitive influence) that are not observed in individual sports.

The present work therefore confirms the value of the individual efforts that the members of a sports team can make to perceive their basic psychological needs as satisfied. These are, at the same time, protective elements that strengthen the vulnerability of the perfectionism–fear of failure relationship. The results invite the applicability of findings in day-to-day training and competitions.

Both coaches and the athletes themselves require psychological involvement that reduces the negative effects and enhances adaptation.

Each aspect, in a coordinated manner in their corresponding role and responsibility, facilitates the work of promoting and providing resources that allow athletes to believe that their decisions and spaces for personal growth are based on autonomy and not on dependence, that learning is part of a greater competence and ability for sports and personal actions, and that they feel that they are in a social space where there is understanding, empathy in the face of mistakes, and unconditional support.

## Figures and Tables

**Table 1:** Descriptive data on sociodemographic variables.

		*n*	%	M_age_	SD_age_
Age (14–19 years)				16.72	3.59
Gender	Boys	194	52.15	16.88	3.82
	Girls	178	47.85	16.13	2.96
Sports	Soccer	97	26.07		
	Handball	96	25.81		
	Volleyball	68	18.28		
	Basketball	111	29.84		
Experience (years)	Less than 2	78	20.97		
	2–5 years	136	36.56		
	More than 5	158	42.47		

Note. *n* = sample; % = percentage; M = mean; SD = standard deviation.

**Table 2:** Descriptive data and correlations of the study variables.

	Range	K-S	M (SD)	1	2	3	4	5	6
1. Strivings for Perfection	1–6	0.43	61.3 (4.23)	(0.93)					
2. Negative Reactions to Imperfection	1–6	0.34	59.6 (3.18)	0.46 **	(0.87)				
3. Autonomy	1–5	0.62	13.4 (2.48)	0.36 **	−0.56 **	(0.84)			
4. Competence	1–5	0.38	12.8 (2.17)	0.28 **	−0.64 **	0.68 **	(0.91)		
5. Social Relation	1–5	0.25	14.1 (2.61)	−0.13 **	−0.48 **	0.63 **	0.69 **	(0.93)	
6. Fear of failure	1–5	0.51	15.7 (2.50)	0.51 **	0.56 **	−0.41 **	−0.38 **	−0.45 **	(0.88)

Note. M: Mean; SD: Standard Deviation; K-S: Kolgomorov–Smirnov; ** < 0.01.

**Table 3:** Partial correlation indexes controlling for the variable types of sport and sporting experience (years).

Type of Sport	1	2	3	4	5	6
1. Strivings to Perfection	-					
2. Negative Reactions to Imperfection	0.48 **	-				
3. Autonomy	0.26 **	−0.53 **	-			
4. Competence	0.37 **	−0.50 **	0.58 **	-		
5. Social Relation	−0.41 **	−0.46 **	0.68 **	0.54 **	-	
6. Fear of Failure	0.47 **	0.58 **	−0.53 **	−0.49 **	−0.50 **	-
**Sporting Experience**	**1**	**2**	**3**	**4**	**5**	**6**
1. Strivings to Perfection	-					
2. Negative Reactions to Imperfection	0.36 **	-				
3. Autonomy	0.49 **	−0.42 **	-			
4. Competence	0.38 **	−0.56 **	0.61 **	-		
5. Social Relation	−0.42 **	−0.49 **	0.54 **	0.57 **	-	
6. Fear of Failure	0.53 **	0.42 **	−0.52 **	−0.45 **	−0.48 **	-

Note. ** < 0.01.

## Data Availability

Data are available to the correspondence author by a reasonable request.
